# Supplementary material for: Myoparr-Associated and -Independent Multiple Roles of Heterogeneous Nuclear Ribonucleoprotein K during Skeletal Muscle Cell Differentiation
Source: Int J Mol Sci. 2021 Dec 22;23(1):108. doi: 10.3390/ijms23010108 (PMC8744952; doi:10.3390/ijms23010108)
Supplement: Supplementary file 1 [file ijms-23-00108-s001.zip › Supplementary Files/Figure S1-4.pdf]

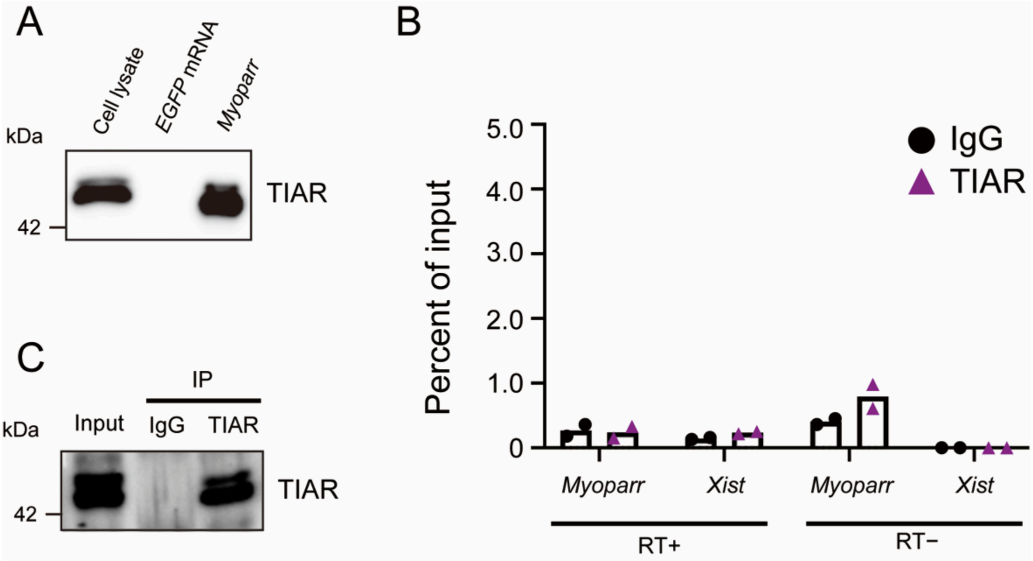

Figure S1. Detection of the interaction between *Myoparr* and TIAR in differentiating C2C12 cells. (A) The interaction between synthesized *Myoparr* and TIAR was detected by immunoblotting using a TIAR antibody. (B) qRT-PCR for the detection of *Myoparr* and *Xist* following RNA-immunoprecipitation using a TIAR antibody. The presence or absence of reverse transcription reaction is shown by (RT+) or (RT−), respectively. Bars indicate the average of two independent experiments, and circles and triangles represent the values of each experiment. (C) Purified TIAR protein from C2C12 cells by immunoprecipitation using a TIAR antibody was confirmed by western blotting.

A

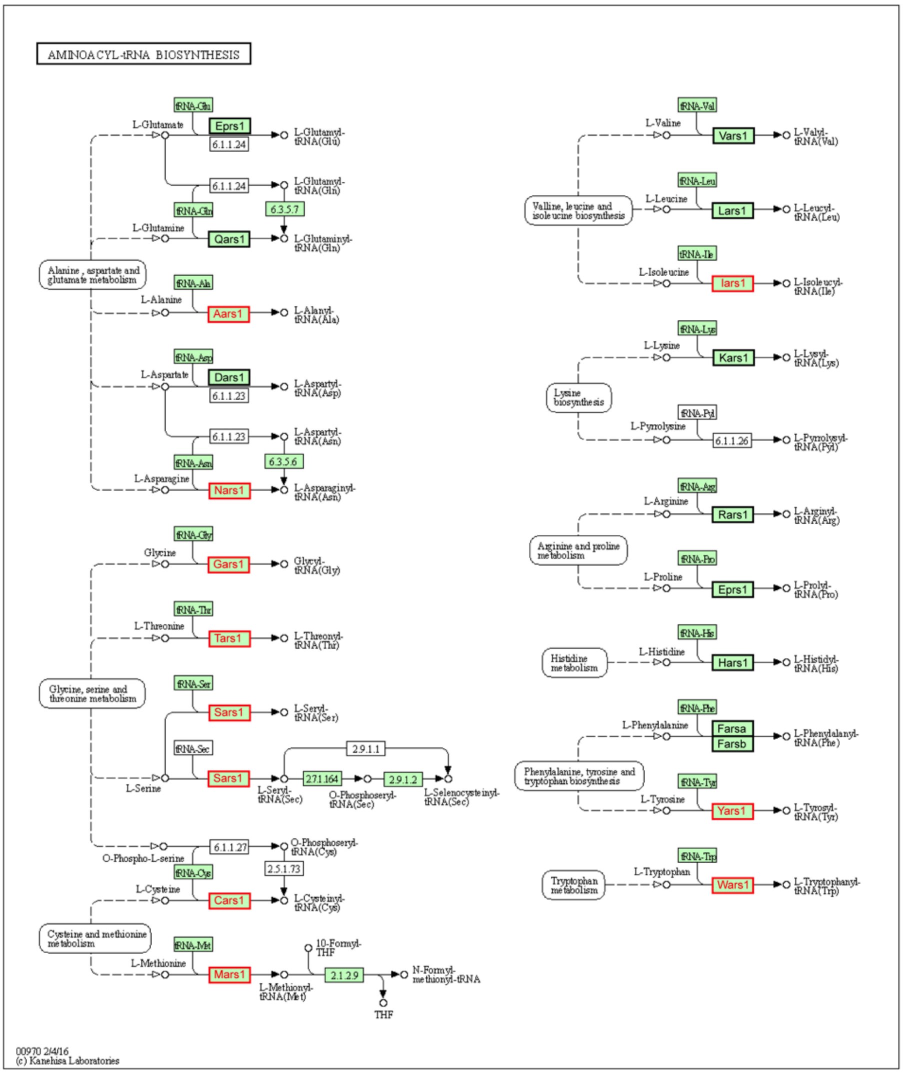

B Cytosolic aminoacyl-tRNA synthetases

Gene Name	Description	log2 Foldchange	padj
Aars (Aars1)	alanyl-tRNA synthetase	1.189688646	6.64E-11
Rars (Rars1)	arginyl-tRNA synthetase	0.717750186	0.000603064
Nars (Nars1)	asparaginyl-tRNA synthetase	1.037402961	1.73E-09
Dars (Dars1)	aspartyl-tRNA synthetase	0.298274648	0.325335115
Cars (Cars1)	cysteinyl-tRNA synthetase	0.872794782	2.21E-05
Qars (Qars1)	glutaminyl-tRNA synthetase	0.081217744	0.864024052
Eprs (Eprs1)	glutamyl-prolyl-tRNA synthetase	0.585228462	0.018216177
Gars (Gars1)	glycyl-tRNA synthetase	1.205275471	3.91E-13
Hars (Hars1)	histidyl-tRNA synthetase	0.413615028	0.200347566
Iars (Iars1)	isoleucine-tRNA synthetase	1.022779598	8.54E-07
Lars (Lars1)	leucyl-tRNA synthetase	0.709821097	0.000114578
Kars (Kars1)	lysyl-tRNA synthetase	0.349181856	0.168606343
Mars (Mars1)	methionine-tRNA synthetase 1	0.900197979	1.01E-05
Farsa	phenylalanyl-tRNA synthetase, alpha subunit	0.200736108	0.620163384
Farsb	phenylalanyl-tRNA synthetase, beta subunit	0.710725176	0.000803764
Sars (Sars1)	seryl-aminoacyl-tRNA synthetase	1.078993838	2.66E-06
Tars (Tars1)	threonyl-tRNA synthetase	0.905000211	8.27E-07
Wars (Wars1)	tryptophanyl-tRNA synthetase	0.878940288	5.01E-06
Yars (Yars1)	tyrosyl-tRNA synthetase	0.772263805	0.000703005
Vars (Vars1)	valyl-tRNA synthetase	0.593311919	0.003371758

C Mitochondrial aminoacyl-tRNA synthetases

Gene Name	Description	log2 Foldchange	padj
Aars2	alanyl-tRNA synthetase 2, mitochondrial	-0.781345711	0.012684624
Rars2	arginyl-tRNA synthetase 2, mitochondrial	0.020716521	0.976825609
Nars2	asparaginyl-tRNA synthetase 2 (mitochondrial)(putative)	0.356286565	0.378947013
Dars2	aspartyl-tRNA synthetase 2 (mitochondrial)	-0.118907946	0.852207722
Cars2	cysteinyl-tRNA synthetase 2 (mitochondrial)(putative)	-0.419664503	0.30297916
Ears2	glutamyl-tRNA synthetase 2, mitochondrial	0.432715891	0.343242224
Hars2	histidyl-tRNA synthetase 2	-0.125988909	0.813766049
Iars2	isoleucine-tRNA synthetase 2, mitochondrial	-0.111305242	0.796505735
Lars2	leucyl-tRNA synthetase, mitochondrial	-0.257452115	0.40655708
Mars2	methionine-tRNA synthetase 2 (mitochondrial)	-0.463741055	0.383638447
Fars2	phenylalanine-tRNA synthetase 2 (mitochondrial)	0.24527022	0.65503804
Pars2	prolyl-tRNA synthetase (mitochondrial)(putative)	0.203237774	0.784806846
Sars2	seryl-aminoacyl-tRNA synthetase 2	-0.782886579	0.039853007
Tars2	threonyl-tRNA synthetase 2, mitochondrial (putative)	0.25526333	0.552363803
Wars2	tryptophanyl tRNA synthetase 2 (mitochondrial)	0.172212967	0.789056335
Yars2	tyrosyl-tRNA synthetase 2 (mitochondrial)	0.078853278	0.90065489
Vars2	valyl-tRNA synthetase 2, mitochondrial	0.188587495	0.706779801

Figure S2. RNA-Seq revealed the altered expression of a group of genes coding for cytosolic aminoacyl-tRNA synthetases by *hnRNPK* KD. (A) The KEGG pathway diagram of cytosolic aminoacyl-tRNA biosynthesis. The gene names surrounded by the red frame indicate genes that are significantly upregulated by *hnRNPK* KD. (B-C) The results of the RNA-Seq analysis showing the altered expression of genes coding for cytosolic aminoacyl-tRNA synthetases (B) and mitochondrial aminoacyl-tRNA synthetases (C) by *hnRNPK* KD. Red and blue indicate genes that are significantly upregulated or downregulated, respectively, by *hnRNPK* KD.

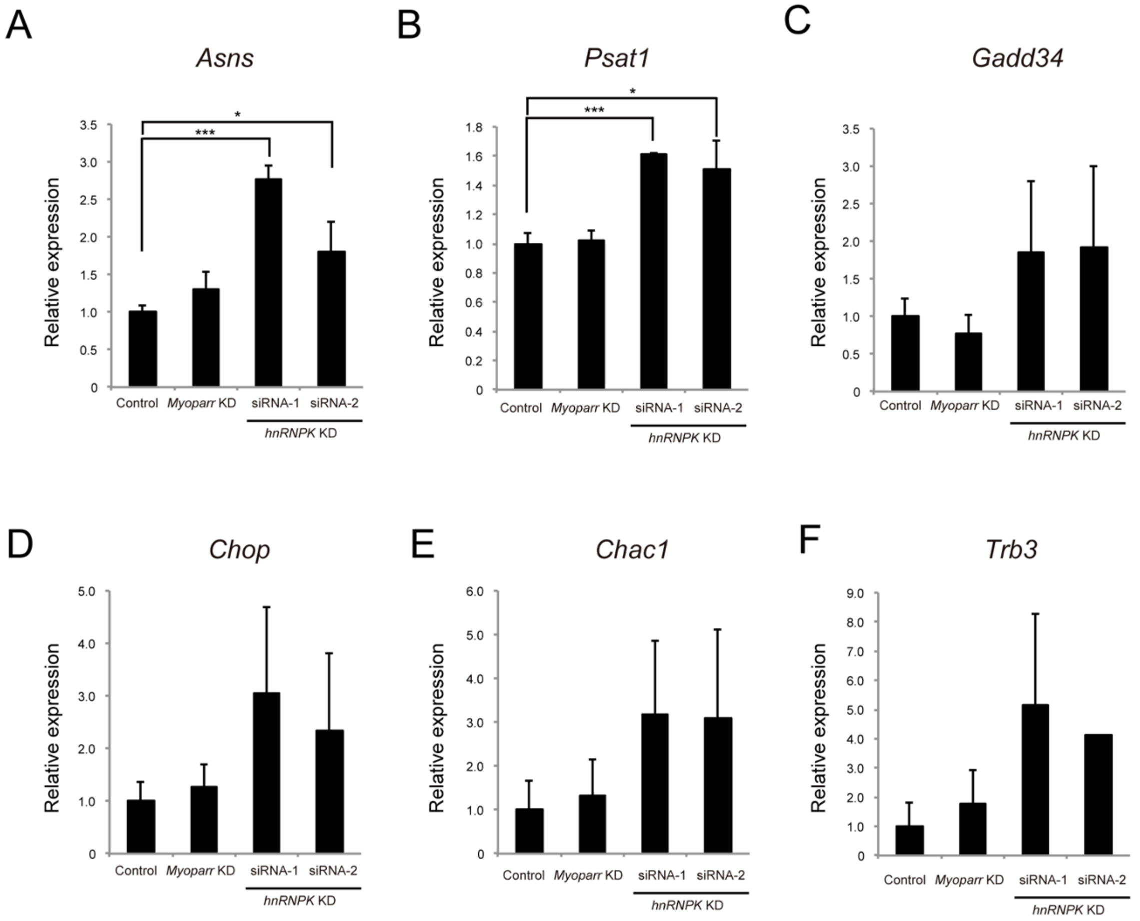

Figure S3. The expression changes of Atf4 target genes by *hnRNPK* KD in differentiating myoblasts. (A-F) The results of qRT-PCR for detecting the expression of *Asns* (A), *Psat1* (B), *Gadd34* (C), *Chop* (D), *Chac1* (E), and *Trb3* (F) 48 h after *hnRNPK* KD. n = 3, mean ± SD. ***$p < 0.001$, *$p < 0.05$.

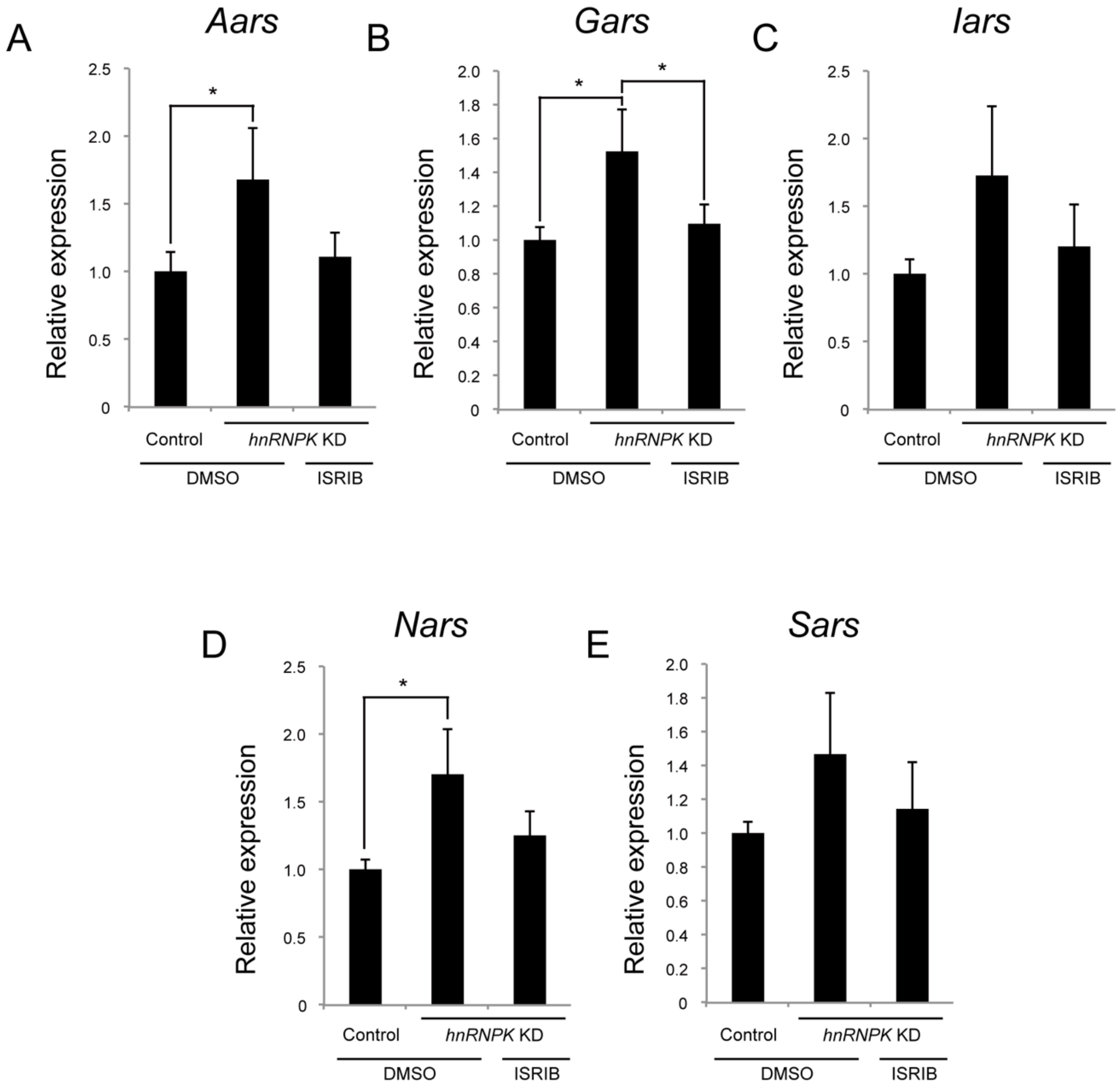

Figure S4. hnRNPK regulates the expression of a group of genes coding for aminoacyl-tRNA synthetases via the eIF2α/Atf4 pathway. (A-E) The results of qRT-PCR for detecting the altered expression of *Aars* (A), *Gars* (B), *Iars* (C), *Nars* (D), and *Sars* (E) 48 h after *hnRNPK* KD (using a different siRNA from Figure 6) with or without ISRIB treatment. n = 3, mean ± SD. *$p < 0.05$.
